# Generation and Characterization of a Novel Recombinant Antibody Against 15-Ketocholestane Isolated by Phage-Display

**DOI:** 10.3390/ijms13044937

**Published:** 2012-04-19

**Authors:** Md. Omedul Islam, Yan Ting Lim, Conrad En Zuo Chan, Amaury Cazenave-Gassiot, J. Ludovic Croxford, Markus R. Wenk, Paul A. Macary, Brendon J. Hanson

**Affiliations:** 1 Immunology Program, Department of Microbiology, Yong Loo Lin School of Medicine, Centre for Life Sciences, National University of Singapore, 117456 Singapore; E-Mails: omedul_islam@nuhs.edu.sg (M.O.I.); ting@nus.edu.sg (Y.T.L.); cenzuo@dso.org.sg (C.E.Z.C.); ludo_von_crox@yahoo.co.uk (J.L.C.); paul_macary@nuhs.edu.sg (P.A.M.); 2 Department of Biochemistry, Yong Loo Lin School of Medicine, Centre for Life Sciences, National University of Singapore, 117456 Singapore; E-Mails: slsiacg@nus.edu.sg (A.C.-G.); bchmrw@nus.edu.sg (M.R.W.); 3 Defence Medical and Environmental Research Institute, DSO National Laboratories, 117510 Singapore, Singapore

**Keywords:** phage display, oxysterols, multiple sclerosis, mass-spectrometry

## Abstract

The employment of monoclonal antibodies (Mabs) to identify disease-associated biomarkers in clinical samples represents the underlying principle for many diagnostic tests. To date, these have been principally developed for protein targets with few reported applications for lipids due to their hydrophobicity and poor immunogenicity. Oxysterols represent a family of lipids implicated in diverse human diseases where Mab-based detection assays could have a profound effect on their utility as clinical biomarkers. These are usually identified in patients’ samples by mass- spectrometry based approaches. Here, we describe an antibody phage-library based screening methodology for generating a recombinant monoclonal antibody (RAb) targeting the oxysterol-15-ketocholestane (15-KA), a lipid implicated in multiple sclerosis and Autoimmune Encephalomyelitis (EAE). The antibody is highly specific for 15-KA and shows little or no binding activity for other closely related oxysterols. We employ RAb2E9 to address the controversy over whether 15-KA is a true biomarker for MS/EAE and show that 15-KA is undetectable in serum taken from mice with EAE using antibody based detection methodologies; a finding confirmed by mass-spectrometry analysis. This study demonstrates the technical feasibility of using phage display to isolate highly specific antibodies against poorly immunogenic, small molecule lipids.

## 1. Introduction

Monoclonal antibodies are routinely employed in biomarker identification and quantitation as vital components of radioimunoassays, enzyme linked immunosorbent assays (ELISA), immunocytopathology and flow cytometry assays for *in vitro* and *in vivo* diagnoses, imaging and immunotherapy [1]. The use of antibodies as diagnostic tools has been limited thus far to mainly protein antigens. For diseases where lipids are implicated and would make ideal biomarkers, the employment of antibodies is restricted to detecting anti-lipid IgGs but not the lipid antigens themselves. The detection of anti-lipid IgGs is associated with autoimmune inflammatory diseases such as systemic lupus erythematosus (SLE), rheumatoid arthritis and systemic sclerosis, where antiphospholipid syndrome (APLS) is a common complication [2]. An antibody-based detection of lipid antigens would potentially form a complementary approach aimed at detecting the relevant lipid-based parameters at an earlier stage in disease than the antibody responses they engender.

Oxysterols are reported to be altered in several neurodegenerative and demyelinating diseases such as Alzheimer’s disease (AD) and multiple sclerosis (MS) [3]. The characterization of oxysterols as *bona fide* ligands for the nuclear receptor, liver x receptor (LXR) [4], has augmented their potential as biomarkers for these common neurodegenerative disorders [5]. Some controversy exist however as to the identification of oxysterol 15-ketocholestane (15-KA) in MS [6]. The involvement of 15-KA in MS was first suggested through serum and cerebrospinal fluid anti-lipid IgG characterization on lipid microarrays [7]. Administration of 15-KA amongst a group of oxidized cholesterol derivatives to mice in the MS animal model experimental autoimmune encephalomyelitis (EAE) exacerbated the disease [8] and detection of a number of oxysterols including 15-KA has been made in both MS and EAE [9]. However, a recent study was unable to support this association in both MS patients and EAE mice [6] and thus the true potential of 15-KA as an MS biomarker remains controversial.

As biological analytes, oxysterols are challenging to measure based on their low abundance against a high background of cholesterol. Mass-spectrometry is the analytical method of choice either in the GC-MS or LC-MS format, but requires extensive sample preparation to help resolve the oxysterols from other more abundant lipids [10–12]; and this has been held responsible for controversy that currently exists over the identification of the 15-KA in MS [13]. Antibody based detection methods with sterol-specific antibodies could overcome these challenges, as this would allow detection in a complex lipid matrix.

We describe here the generation of 15-KA specific antibody to broaden the range of assays that can be utilized to detect and quantify 15-KA. Traditionally, monoclonal antibodies against small lipid or other biomolecules are difficult to generate as they are poor immunogens that do not engender good antibody responses in immunized rodents and often require conjugation to a protein carrier to produce a sufficiently good immune response for hybridoma generation [14,15]. Indeed our efforts to raise antibodies against unconjugated 15-KA using the classical mouse hybridoma techniques were unsuccessful. We therefore selected an antibody-generating methodology that avoided the requirement for generating an immune response, which was based on the *in vitro* screening of a non-immunized recombinant human Fab phage library. From this library, we were able to isolate a RAb specific for 15-KA. This antibody is highly specific for 15-KA and shows little or no binding activity to other closely related oxysterols. Using this antibody, we then sought to address the controversial identification of 15-KA as a biomarker in EAE/MS.

## 2. Results and Discussion

### 2.1. Panning the Fab-phage Library with 15-KA

Previous studies had shown that under pathological conditions such as atherosclerosis, the presentation of oxysterols may differ from the normal physiological state and may coalesce into lipid microcrystals through a cycle of aggregation and inflammation [16]. Murine monoclonal antibodies previously generated against cholesterol have also been shown to bind crystalline forms of the lipid generated by evaporation from solution in ethanol [17]. In light of these previous findings and the difficulty of dissolving a highly non-polar lipid in an aqueous solution, we decided to pan the phage library against 15-KA microcrystals coated onto a solid support immunotube by evaporation from an ethanol solution.

The human phage antibody library underwent 5 rounds of panning with the enriched phage antibodies assayed for binding activity after each panning step. Enrichment for 15-KA-specific polyclonal antibodies was observed from the 4th round of panning onwards. After the final pan against 15-KA, Fabs from 380 single colonies were tested for their ability to bind to 15-KA by ELISA and five clones selected based on their unique combination of heavy and light variable chain sequences as determined by BstN1 restriction digest and Sanger sequencing. The CDR3 amino acid sequences and germline variable genes used are given in Table 1. These five clones were subsequently converted to human IgG1s and expressed in mammalian tissue culture as full length recombinant antibodies.

### 2.2. Specificity of Anti-15-KA Human IgG for Crystalline 15-KA

**Figure 1:**
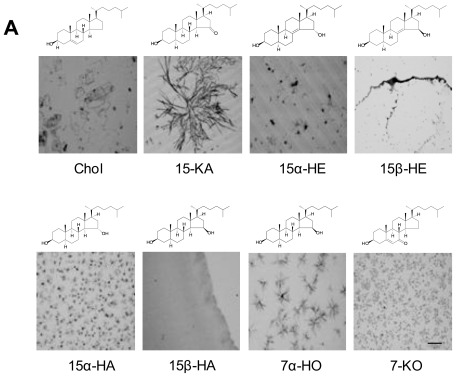

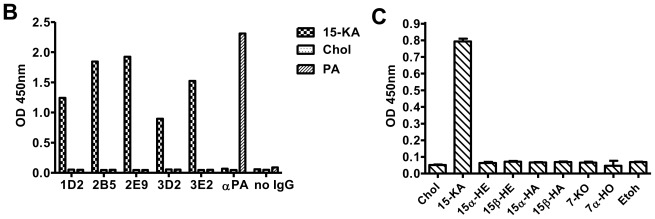
(**A**) Chemical structures of cholesterol, 15-KA, and a selected panel of oxysterols that are closely related to 15-KA. Light micrographs of crystals of pure anhydrous sterols dried from ethanol were taken at magnification of 40X. Scale bar equals 30 μm. (**B**) Analysis of unique RAb isolate from panning against 15-KA following conversion to human IgG1. Coating of the microtitre plates was at 1 μg per well and binding was measured by ELISA. (**C**) Analysis of specificity of antibody RAb2E9. Cholesterol, 15-KA and the indicated panel of oxysterols were coated on microtitre plates at 1μg per well. Reactivity of RAb2E9 to the coated sterols was measured using ELISA.

Given our desire to generate antibodies against 15-KA microcrystals, we carried out microscopic examination of ethanol-evaporated 15-KA to confirm crystal formation. As binding of the antibody may be specific to the type of crystal structure and not the specific oxysterol, we concurrently examined a series of related oxysterols dissolved in ethanol and evaporated in the same manner. Microscopic examination showed that each oxysterol either formed a unique crystalline microstructure or a monolayer after ethanol evaporation (Figure 1A).

Having confirmed the microcrystal structure of 15-KA, the 5 unique IgG were then tested for binding affinity against these oxysteols including 15-KA by ELISA (Figure 1B). All five clones had similar specific binding activity for 15-KA and did not bind any other oxysteol in spite of their close similarity in molecular structure (Figure 1A). We show a representative binding profile of the antibody with the strongest binding for 15-KA (RAb2E9) to this panel of oxysterols including 7-ketocholesterol and 15-ketocholestene (Figure 1C). Thus, the monoclonal human anti-15-KA IgG only binds to a higher order crystalline form of 15-KA but not to the crystalline forms of other oxysterols. This could be due either to the difference in crystal structure or the different molecular structure of the oxysterols or a combination of both.

### 2.3. Sensitivity of Human Anti-15-KA IgG

**Figure 2:**
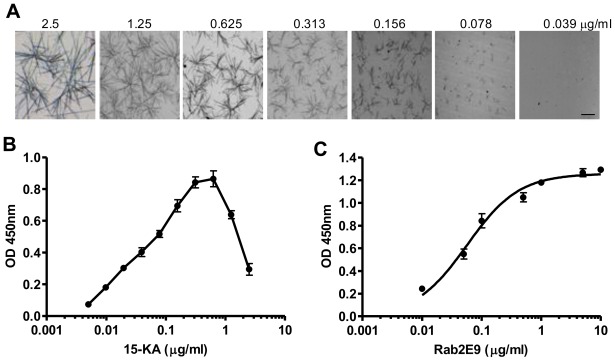
(**A**) Light micrographs of 15-ketocholestane crystals over a concentration range from 2.5 μg/mL to 0.005 μg/mL after coating in 96-microtitre wells. Images were taken at 40 × magnification. Scale bar equals 50 μm. (**B**) Titration of 15-ketocholestane against RAb2E9 using direct ELISA. The plate, which was used for microcrystal imaging, was then processed for ELISA with 1 μg/mL of Rab2E9 antibody. (**C**) Dose dependent binding of RAb2E9 with 15-KA. 15-KA was coated at 20 ng/well and the ELISA performed with the indicated concentrations of RAb2E9.

To determine the sensitivity of the RAb2E9 for 15-KA, the antigen (15-KA) was titrated from 2.5 μg/mL to 0.005 μg/mL and crystal formation observed microscopically (Figure 2A). RAb2E9 exhibited good binding activity across the range of concentrations and was able to detect reliably down to 10 ng/mL using ELISA (Figure 2B). Interestingly, the crystal structure of 15-KA varies with concentration and at 0.156 μg/mL resembles the structure of the oxysterol 7α-hydroxycholesterol (7α-HO) included in the figure 1A (Figure 2A). The fact that RAb2E9 binds 15-KA at this concentration strongly to 15-KA but not 7α-HO suggests that the specificity is due to the differences in oxysterol molecular structure. In addition, the ability of RAb2E9 to bind 15-KA crystals of varying microstructures indicates that crystal structure does not determine antibody specificity. The ability of this antibody to distinguish such minute difference in oxysterol structure (a loss of a keto group on the 15-C and a gain of a hydroxyl on the nearby 7-C) is surprising.

### 2.4. Detection of 15-KA in EAE Mouse Serum with RAb2E9

**Figure 3:**
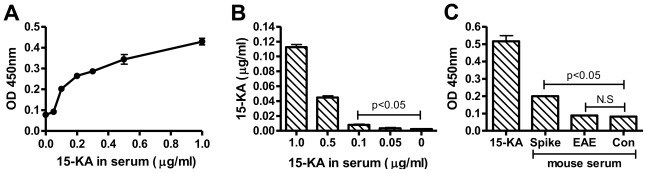
(**A**) Spiking of mouse serum where 15-KA was dissolved in mouse serum at indicated concentrations and mixed overnight, then extracted with cholorofrom:methanol. The extracted samples were coated onto the ELISA plates and detected with RAb2E9 antibody. (**B**) Mass-spectrometry analysis of 15-KA recovered from the spiked samples. (**C**) Lipids from spiked serum, EAE and control mouse blood serum were extracted and analyzed by ELISA with RAb2E9.

It was reported by Farez and colleagues that 15-KA is present in MS patient serum and EAE mouse serum in concentration ranges from 1.2 μg/mL to 300 ng/mL [9]. Based on our *in vitro* analyses, these are clearly within the detection limits of RAb2E9 in ELISA and combined with the antibody’s exquisite specificity should allow detection in serum without the complex manipulations needed for mass spectrometry. To ascertain the feasibility for detecting 15-KA in serum, we tested the detection of 15-KA in murine serum spiked with 15-KA from 1 μg/mL to 50 ng/mL following extraction of the lipid fraction and coating on microtitre plates for ELISA. RAb2E9 was able to detect 15-KA in samples spiked with 1 μg/mL to 100 ng/mL of 15-KA (Figure 3A). Analysis with LC-MS affirmed the recovery of 15-KA from the same samples (down to concentrations of 100 ng/mL) (Figure 3B).

EAE mice immunized with MOG35-55 were monitored for the development of total hind limb paralysis as a marker for severe disease. At this stage, mice were euthanized and blood samples collected by cardiac puncture. Total lipid was extracted from the EAE mouse serum together with spiked and unspiked normal mouse serum as positive and negative control respectively and the presence of 15-KA analyzed by ELISA with RAb2E9 (Figure 3C). Despite the positive signal in the spiked sample, 15-KA was not detected in the EAE mouse serum. Moreover, similar to the study performed by Bjorkhem and colleagues [6], no detectable levels of 15-KA were found by LC-MS (data not shown).

### 2.5. Discussion

With the important exception of glycolipids, relatively few antibodies that recognize specific lipids have been described. Little is known about the precise molecular requirement for successful generation of antibodies against lipids either in terms of their presentation during immunization *in vivo* or their selection *in vitro*. However, with the increasing availability of pure, synthetic, and stable lipids, we now have the opportunity to systematically screen these stable antigens against a non-immune phage library. As the phage display technique bypasses the use of animals and requires a shorter time frame relative to the hybridoma approach, it could be used to generate a whole class of novel tools for basic research and medical diagnostics with applications in immunohistochemistry, cytochemistry and biochemistry. In this study, we show that it is possible to generate antibodies against non-immunogenic oxysterols using phage display, bypassing the need of conjugating the oxysterol to immunogenic epitopes or using adjuvants to enhance its immunogenicity.

We characterized the monoclonal anti-15-KA antibody, RAb2E9, for its antigen-binding properties and found higher binding for crystalline 15-KA, suggesting its binding site is a three-dimensional molecular arrangement on the surface of the oxysterol crystal. This binding to the crystal could be via stereoselective supramolecular interactions with tertiary structural motifs that form part of the higher molecular structure of the 15-KA crystal. We can speculate that the epitope is governed by the position of the double bond and the ketone group within the B and D-ring of the sterol, as the antibody is specific for 15-KA and not for other oxysterols (with modification in the double bonds hydroxyl group) closely related to 15-KA. The RAb2E9 antibody exhibited a detection limit of 10 ng/mL for pure crystalline 15-KA, and 100 ng/mL for 15-KA extracted from spiked plasma samples. A few groups have generated antibodies against crystalline cholesterol using the classical hybridoma approach developed by Köhler and Milstein [17,18], but only antibodies of the IgM isotype were generated due to the non-immunogenic nature of the antigen. However, our use of a Fab phage library allowed for engineering of the isotype of the fragment crystallizable region (Fc region) to IgG.

Several groups have studied 15-KA in the context of multiple sclerosis (MS). Kanter *et al.* identified anti-15-KA autoantibody from the cerebrospinal fluid derived from relapsing MS patients, and Farez *et al.* were able to detect 300 ng/mL of 15-KA in the serum of mice with EAE using LC-MS [7,9]. This finding has since been challenged, as separate studies were unable to verify the presence of oxysterols in MS patients’ sera relative to healthy controls and concluded that if present at all it would only be in trace amounts [6,13]. Discussion centered on this controversy has suggested that the need for extensive sample preparation to allow reduction of the cholesterol may be a cause of the discrepancy [13]. As RAb2E9 does not react to other oxysterols or cholesterol and has a functional range straddling the 15-KA concentration suggested to be present, we were able to perform analysis in crude lipid extracts of EAE serum without the need for excessive sample preparation. We could not detect 15-KA above that present in normal mouse serum with our antibody, a result we confirmed with mass-spectrometry. Given our limit of detection, this contrasts with the findings of Farez *et al.* [9]. Hence we are unable to confirm a role for 15-KA as a biomarker in EAE, the mouse model of MS.

## 3. Experimental Section

### 3.1. Materials

Cholesterol (Chol) and its derivatives, 15-ketocholestane (15-KA), 15α-hydroxycholestane (15α-HA), 15β-hydroxycholestane (15β-HA), 15α-hydroxycholestene (15α-HE), 15β-hydroxycholestene (15β-HE), 15-ketocholestene (15-KE), 7-ketocholesterol (7-KO) and 7α-hydroxycholesterol (7α-HO) were purchased from Avanti Polar Lipids, Inc (Alabama, USA). Goat anti-human (H+L chain) horseradish peroxidase (HRP) conjugated secondary antibody was obtained from (Thermo Scientific Pierce, USA), ELISA plates and immunotubes from NUNC (Denmark). Anti M13-HRP conjugated secondary antibody was purchased from Pharmacia (Upsala, Sweden). All other analytical grade chemicals were obtained from Sigma (St. Louis, MO, USA).

### 3.2. Fab-phage Library Screening

Library screening was performed using the non-immune human Fa- phage display library HX01 (Humanyx Pte Ltd, Singapore) displayed in the pCES1 vector [19]. To prepare immunotubes for panning, 15-KA was coated by adding the lipid in ethanol (200 μg/mL) and allowing the solvent to evaporate while the immunotubes were mixed horizontally overnight at room temperature. The coated immunotubes were then blocked in 2% skim milk (SM) in PBS for 1 h at room temperature prior to panning. Screening of the library was performed on antigen coated immunotubes essentially as described previously for protein antigens [20]. Briefly, Fab-phage were incubated with coated immunotubes for 2 h at room temperature, followed by washing with PBS-T (0.1% Tween-20) to remove non-specific bound phage; specifically bound phage were then eluted by digestion with trypsin solution for 30 min at 37 °C. Stringency of washing was increased during subsequent pans. Following six rounds of panning, enrichment was established using ELISA with the polyclonal Fab-phage preparations from each pan and individual clones were analyzed from the eluted phage of the pan showing the highest level of enrichment. To assess the uniqueness of positive clones, BstN1 restriction digest was performed following PCR amplification of the Fab coding region and resolved on 3% agarose gel. Clones with similar patterns were grouped and the clone with the highest ELISA signal was converted to IgG for subsequent characterization.

### 3.3. Conversion of Fab-phage to Human IgG1

Distinct Fab-phage were converted to IgG1 by cloning the Fab containing fragment from pCES1 into pCMV based vectors harboring the human constant regions for either the lambda or kappa light chain and IgG1 heavy chain separated by an internal ribosome entry sequence [21]. Constructs encoding the Fab-IgGs were transfected into human embryonic kidney (HEK293) cells by use of lipofectamine 2000 (Invitrogen). Culture media was collected 72 h post transfection and fresh media was added to the cells and allowed to incubate for a further 72 h before collection. Secreted antibodies were purified using recombinant Protein-A Sepharose beads (Thermo Scientific Pierce, USA). Specificity of the antibodies was ascertained using ELISA with 15-KA, cholesterol and other oxysterols.

### 3.4. Oxysterol Crystal Imaging and ELISA

Microtitre wells were coated with sterols dissolved in ethanol at the indicated concentration and the solvent allowed to evaporate overnight. For crystal imaging, images were then captured by light microscopy. After imaging the wells were then blocked with 2% SM in PBS for 1 h at room temperature. 100 μL of the indicated antibodies were then added for 2 h, followed by HRP-conjugated goat anti-human IgG (1:5000) in blocking buffer (1 h, room temperature). The reaction was visualized by the addition of 50 μL of chromogenic substrate TMB for 15 min, and was stopped with 100 μL of H_2_SO_4_. Absorbance was measured at 450nm. Plates were washed thrice with PBST between steps.

### 3.5. Lipid Extraction from 15-KA Spiked and EAE Mouse Serum

To produce spiked serum samples, 15-KA was dissolved in DMSO, then added to the serum harvested from naïve mice by cardiac puncture to reach the indicated concentrations and incubated overnight with mixing at 4 °C. To produce EAE mouse serum, mice were injected subcutaneously with 100 mg MOG35–55 and 1 mg heat-killed *Mycobacterium tuberculosis* H37RA (Invitrogen, Carlsbad, CA, USA) emulsified in complete Freund’s adjuvant. Pertussis toxin (200 ng in PBS; List Biological Laboratories, USA) was injected intra-peritoneally on days 0 and 2 after immunization. EAE clinical symptoms were assigned scores daily as follows: 0, no clinical signs; 1, loss of tail tonicity; 2, impaired righting reflex; 3, partial hindlimb paralysis; 4, total hindlimb paralysis as described [22]. EAE mouse blood was collected via cardiac puncture and serum collected via centrifugation. The Institutional Animal Care and Use Committee, National University of Singapore approved all experimental protocols on mice (IACUC number 018/11). The lipid fraction from serum was extracted using the standard chloroform:methanol extraction method as described [23], and diluted in 200 μL chloroform:methanol for LC-MS. The organic phase was collected and dried using a Speedvac. The extracted lipids were dissolved in 100 μL of ethanol for analysis by ELISA.

### 3.6. Analysis of 15-KA Content Using High Performance LC-MS

High performance liquid chromatography analysis was undertaken on an Accela system (Thermo Scientific, USA). Mass spectrometry analysis was undertaken on a LTQ Orbitrap XL instrument (Thermo Scientific) fitted with an electrospray ionisation source (ESI). Due to the unavailability of deuterated 15-KA, internal calibration was not possible. A calibration curve was therefore established by injection of 15-KA standard (Avanti Polar Lipids) at concentrations ranging from 10 ng/mL to 50 μg/mL. This calibration curve was subsequently use for quantification of 15-KA in the samples.

Standards and dried lipid extract samples were dissolved in chloroform:methanol 1:1 (v:v) and 20 μL was used for LC on a Zorbax Eclipse XDB-C18 column (Agilent Technologies) as described previously [24]. Mass spectrometry parameters were as follows: source voltage 3.8 kV, capillary voltage 36 V, tube lens 80, capillary temperature 300 °C, sheath gas flow 40, auxiliary gas flow 10. The two main ions observed with 15-KA are at m/z 385.3 ([M + H-H_2_O]^+^) and 403.4 ([M + H]^+^), these two ions were monitored using single reaction monitoring scans for 15-KA detection in the samples.

## 4. Conclusions

In this study we have shown the development of a RAb against the oxysterol 15-KA, a lipid implicated in MS/EAE, using a phage-library based screening methodology. RAb2E9 exhibits little or no binding to closely related oxysterols and cholesterol, demonstrating its high specificity. We have also addressed the controversy over whether 15-KA is a true biomarker for MS/EAE using RAb2E9 and shown that 15-KA is undetectable in serum taken from mice with EAE using antibody based detection methodologies; a finding confirmed by mass-spectrometry analysis. We are thus the first to report the generation and characterization of human monoclonal antibodies of the IgG_1_ isotype against an oxysterol 15-ketocholestestane; demonstrating the potential of Fab-phage display technique to generate antibodies against crystalline forms of otherwise non-immunogenic lipids, such as oxysterols. This demonstrates the technical feasibility of using phage display to isolate highly specific antibodies against poorly immunogenic, small molecule lipids.

## Figures and Tables

**Table 1:** Sequences of CDR3 for Fab of phage isolated against 15-KA.

Clone	V_L_ CDR3	V_L_ gene	V_H_ CDR3	V_H_ gene
1D2	GTHWPYTFGQG	KV2D-28	GVVGSMDV	HV1-18
2B5	ALQSPFTFGPG	KV2D-28	PPDNRGFYFDF	HV1-3
2E9	GTHWPYTFGQG	KV2D-28	ERAVTNYYYYYGMDV	HV1-3
3D2	ALQTPSFGGG	KV2D-28	ETYSSSWYAPKYFDY	HV1-3
3E2	ALRSPSFGPG	KV2D-28	TPRWLQNIPNDY	HV3-21

## References

[1] Waldmann T.A. (1991). Monoclonal antibodies in diagnosis and therapy. Science.

[2] McNeil H.P., Chesterman C.N., Krilis S.A. (1991). Immunology and clinical importance of antiphospholipid antibodies. Adv. Immunol.

[3] Leoni V. (2009). Oxysterols as markers of neurological disease—A review. Scand. J. Clin. Lab Invest.

[4] Gilardi F., Viviani B., Galmozzi A., Boraso M., Bartesaghi S., Torri A., Caruso D., Crestani M., Marinovich M., de Fabiani E. (2009). Expression of sterol 27-hydroxylase in glial cells and its regulation by liver X receptor signaling. Neuroscience.

[5] Jeitner T.M., Voloshyna I., Reiss A.B. (2011). Oxysterol derivatives of cholesterol in neurodegenerative disorders. Curr. Med. Chem.

[6] Bjorkhem I., Lovgren-Sandblom A., Piehl F., Khademi M., Pettersson H., Leoni V., Olsson T., Diczfalusy U. (2011). High levels of 15-oxygenated steroids in circulation of patients with multiple sclerosis: fact or fiction?. J. Lipid Res.

[7] Kanter J.L., Narayana S., Ho P.P., Catz I., Warren K.G., Sobel R.A., Steinman L., Robinson W.H. (2006). Lipid microarrays identify key mediators of autoimmune brain inflammation. Nat. Med.

[8] Quintana F.J., Farez M.F., Viglietta V., Iglesias A.H., Merbl Y., Izquierdo G., Lucas M., Basso A.S., Khoury S.J., Lucchinetti C.F., Cohen I.R., Weiner H.L. (2008). Antigen microarrays identify unique serum autoantibody signatures in clinical and pathologic subtypes of multiple sclerosis. Proc. Natl. Acad. Sci. USA.

[9] Farez M.F., Quintana F.J., Gandhi R., Izquierdo G., Lucas M., Weiner H.L. (2009). Toll-like receptor 2 and poly(ADP-ribose) polymerase 1 promote central nervous system neuroinflammation in progressive EAE. Nat. Immunol.

[10] Bjorkhem I., Diczfalusy U. (2002). Oxysterols: Friends, foes, or just fellow passengers?. Arterioscler. Thromb. Vasc. Biol.

[11] Dzeletovic S., Breuer O., Lund E., Diczfalusy U. (1995). Determination of cholesterol oxidation products in human plasma by isotope dilution-mass spectrometry. Anal. Biochem.

[12] Quehenberger O., Armando A.M., Brown A.H., Milne S.B., Myers D.S., Merrill A.H., Bandyopadhyay S., Jones K.N., Kelly S., Shaner R.L. (2010). Lipidomics reveals a remarkable diversity of lipids in human plasma. J. Lipid. Res.

[13] Griffiths W.J., Wang Y. (2011). Are 15-oxygenated sterols present in the human circulation?. J. Lipid Res.

[14] Boullerne A., Petry K.G., Geffard M. (1996). Circulating antibodies directed against conjugated fatty acids in sera of patients with multiple sclerosis. J. Neuroimmunol.

[15] Singh K.V., Kaur J., Varshney G.C., Raje M., Suri C.R. (2004). Synthesis and characterization of hapten-protein conjugates for antibody production against small molecules. Bioconjug. Chem.

[16] Biro A., Cervenak L., Balogh A., Lorincz A., Uray K., Horvath A., Romics L., Matko J., Fust G., Laszlo G. (2007). Novel anti-cholesterol monoclonal immunoglobulin G antibodies as probes and potential modulators of membrane raft-dependent immune functions. J. Lipid Res.

[17] Swartz G.M., Gentry M.K., Amende L.M., Blanchette-Mackie E.J., Alving C.R. (1988). Antibodies to cholesterol. Proc. Natl. Acad. Sci. USA.

[18] Perl-Treves D., Kessler N., Izhaky D., Addadi L. (1996). Monoclonal antibody recognition of cholesterol monohydrate crystal faces. Chem. Biol.

[19] De Haard H.J., van Neer N., Reurs A., Hufton S.E., Roovers R.C., Henderikx P., de Bruine A.P., Arends J.W., Hoogenboom H.R. (1999). A large non-immunized human Fab fragment phage library that permits rapid isolation and kinetic analysis of high affinity antibodies. J. Biol. Chem.

[20] Chan C.E., Chan A.H., Lim A.P., Hanson B.J. (2011). Comparison of the efficiency of antibody selection from semi-synthetic scFv and non-immune Fab phage display libraries against protein targets for rapid development of diagnostic immunoassays. J. Immunol. Methods.

[21] Lim A.P., Chan C.E., Wong S.K., Chan A.H., Ooi E.E., Hanson B.J. (2008). Neutralizing human monoclonal antibody against H5N1 influenza HA selected from a Fab-phage display library. Virol. J.

[22] Croxford J.L., Miyake S., Huang Y.Y., Shimamura M., Yamamura T. (2006). Invariant V(alpha)19i T cells regulate autoimmune inflammation. Nat. Immunol.

[23] Bligh E.G., Dyer W.J. (1959). A rapid method of total lipid extraction and purification. Can. J. Biochem. Physiol.

[24] Shui G., Guan X.L., Low C.P., Chua G.H., Goh J.S., Yang H., Wenk M.R. (2010). Toward one step analysis of cellular lipidomes using liquid chromatography coupled with mass spectrometry: application to Saccharomyces cerevisiae and Schizosaccharomyces pombe lipidomics. Mol. Biosyst.

